# Ranking the impact of human health disorders on gut metabolism: Systemic lupus erythematosus and obesity as study cases

**DOI:** 10.1038/srep08310

**Published:** 2015-02-06

**Authors:** David Rojo, Arancha Hevia, Rafael Bargiela, Patricia López, Adriana Cuervo, Sonia González, Ana Suárez, Borja Sánchez, Mónica Martínez-Martínez, Christian Milani, Marco Ventura, Coral Barbas, Andrés Moya, Antonio Suárez, Abelardo Margolles, Manuel Ferrer

**Affiliations:** 1 Centro de Metabolómica y Bioanálisis (CEMBIO), Facultad de Farmacia, Universidad CEU San Pablo, Campus Montepríncipe, Madrid; 2 Department of Microbiology and Biochemistry of Dairy Products, Dairy Research Institute (IPLA), Consejo Superior de Investigaciones Científicas (CSIC), Villaviciosa, Asturias, Spain; 3 Institute of Catalysis, CSIC, Madrid, Spain; 4 Immunology Area, Department of Functional Biology, University of Oviedo, Asturias, Spain; 5 Physiology Area, Department of Functional Biology, University of Oviedo, Asturias, Spain; 6 Department of Life Sciences, Laboratory of Probiogenomics, University of Parma, Parma, Italy; 7 Unidad Mixta de Investigación en Genómica y Salud de la Fundación para el Fomento de la Investigación Sanitaria y Biomédica de la Comunitat Valenciana (FISABIO) and Instituto Cavanilles de Biodiversidad y Biología Evolutiva de la Universitat de València, València, Spain; 8 CIBER en Epidemiología y Salud Pública (CIBEResp), Madrid, Spain; 9 Department of Biochemistry and Molecular Biology, Biomedical Research Centre, University of Granada, Granada, Spain

## Abstract

Multiple factors have been shown to alter intestinal microbial diversity. It remains to be seen, however, how multiple collective pressures impact the activity in the gut environment and which, if any, is positioned as a dominant driving factor determining the final metabolic outcomes. Here, we describe the results of a metabolome-wide scan of gut microbiota in 18 subjects with systemic lupus erythematosus (SLE) and 17 healthy control subjects and demonstrate a statistically significant difference (*p* < 0.05) between the two groups. Healthy controls could be categorized (*p* < 0.05) based on their body mass index (BMI), whereas individuals with SLE could not. We discuss the prevalence of SLE compared with BMI as the dominant factor that regulates gastrointestinal microbial metabolism and provide plausible explanatory causes. Our results uncover novel perspectives with clinical relevance for human biology. In particular, we rank the importance of various pathophysiologies for gut homeostasis.

Our commensal microbiota is a plastic “organ” comprised of trillions of microbes with symbiotic functional capabilities that directly affect human health. Important studies on the relationship of intestinal microbiota with diseases have linked profound changes in the composition of the population and metabolic functions of the gut microbiota to common human intestinal disorders, such as obesity^1^, Crohn's disease and colitis-associated colorectal carcinoma^2^, ulcerative colitis and irritable bowel syndrome^1^, and *Clostridium difficile*-associated diarrhea^3^,^4^. Recent studies have also suggested that factors, such as antibiotic treatments^4^,^5^ and diet^6^, and subject characteristics, such as age^7^, may be involved in alterations in the microbiota. Researchers are beginning to recognize and understand the short- and long-term consequences of these changes^5^,^7^,^8^,^9^.

Although evidence has suggested an additional link between gut microbiota and immune disorders^10^, this relationship remains incompletely understood. In a previous work, a relevant intestinal dysbiosis was described in the prototypical auto-immune disease systemic lupus erythematosus (SLE)^11^. This microbial imbalance was characterized by increased *Bacteroidetes* levels and a lower *Firmicutes*/*Bacteroidetes* ratio. Metagenome functional inference highlighted putative metabolic processes that were potentially associated with SLE patients, such as an overrepresentation of glycan metabolism and oxidative phosphorylation. However, although this *in silico* analysis could be correlated with a higher abundance of some specific bacterial groups in lupus (i.e., *Bacteroidetes*), experimental evidence of the overall gut microbiota functionality in SLE patients is lacking. In this report, a metabolome-wide scan of the gut microbiota in patients (*n* = 18; SLE codes) with the prototypical auto-immune disease SLE^12^ and healthy controls (HC codes; *n* = 17) is presented. The aim was to discuss primarily whether SLE plays a role in shaping the metabolism of gastrointestinal microbiota, and if so, to obtain information on the cause and effect relationship between the altered microbial metabolites and the underlying disease. This study should be of relevance because patients with SLE exhibit a marked predisposition to metabolic syndrome^13^, atherosclerosis^14^, renal and urinary dysfunctions^13^,^15^, and insulin resistance^15^, and some of these disorders have been linked to gastrointestinal microbiota^16^,^17^. These results demonstrate a separation between the chemical compositions of gut microbiota of both the SLE and HC groups, which was not observed by examining the taxa abundance and composition of their corresponding microbiota. In addition, body mass index (BMI) was shown to have a remarkable effect in healthy subjects although having no effect in patients with SLE, which suggests that the auto-immune response is a stronger driver of intestinal dysbiosis than obesity.

## Results

### Study cohorts

SLE patients (*n* = 18) were recruited from the updated Asturian Register of Lupus. All patients fulfilled at least four of the American College of Rheumatology criteria for SLE^11^. All were women of Caucasian descent (49.1 ± 9.7 years old) with no active disease at the time of sampling (Systemic Lupus Erythematosus Disease Activity Index (SLEDAI) score ≤8). Information on the clinical manifestations of the disease was obtained from the individual clinical records (Supplementary Table 1). Only individuals who had not used antibiotics, glucocorticoids, immunosuppressive drugs, monoclonal antibodies, or other immunotherapies in the 6 months prior to enrollment were recruited for the study. Seventeen gender- and age-matched healthy controls (HC codes) were recruited from the same population. To reduce the possibility that our assay was affected by factors known to influence the gut microbial profile, such as age, diet, and medications, patients with similar factors were selected. These factors included gender (all study subjects were women); age; the absence of antibiotics, steroids, and immunological treatments; medical history (presenting with a wide variety of clinical SLE manifestations); disease duration (2–24 years); and an absence of flares of disease activity at the time of sampling (Supplementary Table 1). Additional factors also included the mean dietary intake of energy, macronutrients, micronutrients, fiber, and phyto compounds (Supplementary Table 2) and lifestyle-related factors (smoking, alcohol consumption, physical activity, and use of vitamin and mineral supplements). The complete datasets together with body mass index (BMI) values for each of the investigated individuals are presented in Supplementary Table 3.

### Contribution and prevalence of SLE in gut homeostasis

**Figure 1:**
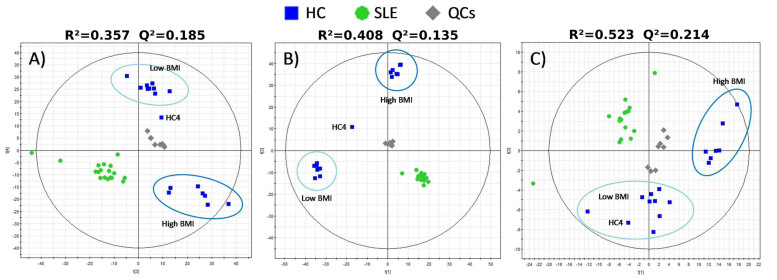
A principal components analysis (PCA) plot for the models built using the set of filtered data that were present in at least 50% of the quality controls (QCs) and for which the coefficients of variation were less than 30% across the QCs. A) PCA plot based on LC-MS (+) data: 4 components (PC3 vs. PC4 shown; no biological variation described by the first two components). B) PCA plot based on LC-MS (−) data: 5 components (PC1 vs. PC2 shown). C) PCA plot based on CE-MS data: 6 components (PC1 vs. PC2 shown). Statistics (R^2^ and Q^2^) are provided in the Figure panels. HC4, with a BMI of 26.29 kg/m^2^, located within “High” and “Low” BMI groups was rejected prior to statistical analysis. The “High BMI” cluster was formed from the following samples (BMI in kg/m^2^ in parentheses): HC6 (27.19), HC8 (27.40), HC19 (25.24), HC20 (28.82), HC26 (30.92), HC29 (36.90), and HC33 (25.95). The “Low BMI” cluster was formed from the following samples: HC11 (20.19), HC13 (24.83), HC14 (24.80), HC16 (22.18), HC21 (23.18), HC22 (23.07), HC28 (21.90), HC30 (22.68), and HC32 (23.13).

Our study protocol comprised the isolation of metabolites from microorganisms obtained from fecal material followed by a metabolome-wide scan via a combination of mass spectrometry (MS) with liquid chromatography (LC) and capillary electrophoresis (CE) separations. Of 134,312 masses, a total of 955 (LC-MS positive mode: 331; LC-MS negative mode: 549; CE-MS: 75) fulfilled statistical criteria for selection (*p* value <0.05; Mann-Whitney *U* test or *t*-test; Supplementary Table 4). A scatter plot based on principal component analysis scores obtained from this set of compounds revealed a clear separation between the SLE and HC groups (Figure 1). Mass signals were highly similar in all SLE patients regardless of age, BMI, disease duration, dietary intake, lifestyle-related factors, or medical history (Supplementary Tables 1–3). Interestingly, the HC group was divided into two distinct sub-groups (Figure 1). A total of 572 of the 134,312 masses (LC-MS positive mode: 155; LC-MS negative mode: 352; CE-MS: 65) caused this separation (*p* < 0.05; Supplementary Table 4). Regardless of age, dietary intake and lifestyle-related factors (Supplementary Tables 2 and 3), HC subjects with BMIs ranging from 20.19 to 24.83 kg/m^2^ formed Cluster 1, whereas those with BMIs ranging from 25.24 to 36.92 kg/m^2^ formed Cluster 2. This separation was not observed in SLE patients (Figure 1), whose BMIs ranged from 19.95 to 37.91 kg/m^2^ (Supplementary Table 3).

Taken together, we demonstrate that the immune status of SLE patients is thus a dominant factor that swiftly regulates the metabolome of the gut microbiota regardless of environmental or individual characteristics. In contrast, in healthy subjects in whom no stronger pressure than BMI exists, BMI (e.g., overweight or obesity) becomes a driving factor determining microbiotal metabolism. The results provided in Figure 1 suggest that the division of the clusters in HC subjects occur within a BMI interval ranging from 24.83 to 25.24 kg/m^2^, which corresponds to the upper and lower limits of the two identified clusters (all samples with BMI ≤ 24.83 kg/m^2^ clustered together, and samples with BMI ≥ 25.24 kg/m^2^ formed a separate cluster). Note that lean-normal individuals are typically characterized by a BMI ≤ 24.99 kg/m^2^, over-weight individuals by a BMI ≥ 25 kg/m^2^ (from 25.0 to 29.99), and obese individuals by a BMI ≥ 30 kg/m^2^ (from 30.0 to 40)^18^. Therefore, one could assume that an unknown mechanism triggers the alteration of gut microbiota functionality at the frontier between lean and over-weight host status. This finding is supported by our previous study in a different population of healthy lean and obese individuals showing that the gut microbiota boosts glycosyl hydrolase activities at a similar BMI range (i.e., 24.5–25 kg/m^2^)^17^. This result is of particular importance as, to the best of our knowledge, no previous investigation has linked host BMI to gut microbiota metabolic dynamics in the development of obesity.

**Figure 2:**
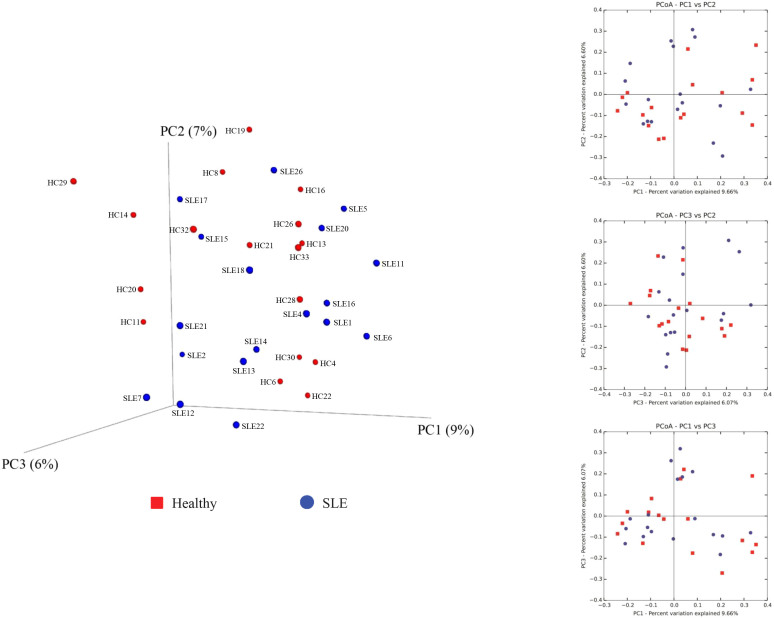
Principal coordinate analysis (PCoA) of SLE and HC based on taxa abundance and composition of gut microbiota. Three-dimensional and bi-dimensional PCoA representation based on a UniFrac similarity matrix of the composition of SLE and HC sample operation taxonomic units (OTUs) is shown. Percentages shown along the axes represent the proportion of dissimilarities captured by the axes. Total bacterial 16S rRNA data and taxa abundance were determined as reported^11^. The raw sequences reported in this article have been deposited in the NCBI Short Read Archive (SRA) (study accession number: SRP028162). Codes are as shown in Supplementary Table 3.

It should be highlighted that a principal coordinate analysis (PCoA) of SLE and HC subjects, based on 16 rRNA microbiota profiles, did not result in subcategorized clusters, either between SLE and HC or between HC subjects with high and low BMI (Figure 2)^11^. This suggests that the changes induced by SLE or BMI (in healthy controls) become marked at the highest level of the functional hierarchy, i.e., the metabolite level (Figure 1), regardless of the heterogeneities that appear below the functional level, i.e., the level of microbiotal population structure (or 16S rRNA).

### Association of SLE with chemical compositions in the gut microbiota: explanatory analysis

As mentioned above, only 955 of 134,312 (or 0.72% of the total) mass features statistically (*p* < 0.05) differed between the SLE and HC groups, suggesting that the impact of SLE on the gut microbiotal metabolite-wide flux distribution and on metabolism itself is moderately low. As we are aware that our study identifies metabolic signatures (955) associated with immune status in SLE compared to HC individuals, mechanisms explaining these associations must be proposed. For this purpose, empirical formulas were assigned to masses that achieved statistical criteria (*p* < 0.05) with a maximum error of 5 ppm using the CEU Mass Mediator (http://biolab.uspceu.com/mediator). We describe each of the major effects linked to key chemical species below.

We first observed that SLE patients exhibited reduced levels of homoserine lactone (HSL) (11.3-fold reduction; *p* = 0.001; Supplementary Table 5). HSL is the degradation product of *N*-acyl-HSLs (AHSL) when metabolized by AHSL lactonases and acylases^19^,^20^,^21^. The reduced accumulation of HSL in SLE patients may be related to an increase in the activity of quorum quenching enzymes that can decrease the pool of AHSL and thus might attenuate quorum sensing and cell-to-cell communication and promote disease progression^19^,^20^,^21^,^22^,^23^.

Compared to HC subjects, SLE patients also exhibited significantly reduced levels of N-acetylmuramic acid (MURNAc) (25.0-fold reduction; *p* = 0.00005) and, to a lesser extent, N-acetylglucosamine (1.5-fold reduction; *p* = 0.0004) (Supplementary Table 5). Both are essential components of the peptidoglycan biopolymer of bacterial cell walls. Peptidoglycans have long been known to promote an inflammatory response; thus, lowering the production of peptidoglycan components, caused by deficiencies in key enzymes^24^ and/or the bacteria that express them, has been demonstrated to potentially influence signaling, disease factors and immune responses^16^,^25^,^26^. In addition, a series of observations have led to the hypothesis that in patients with rheumatoid arthritis with a genetic basis, normal intestinal microbiota harbor bacteria with cell walls capable of stimulating rheumatoid factor, thus possibly inducing arthritis^16^. It is therefore plausible that SLE induces deficiencies in signaling chemical species, particularly MURNAc, that compose the cell walls of gastrointestinal bacteria and that these deficiencies affect the progression of the disease and its collateral effects.

**Figure 3:**
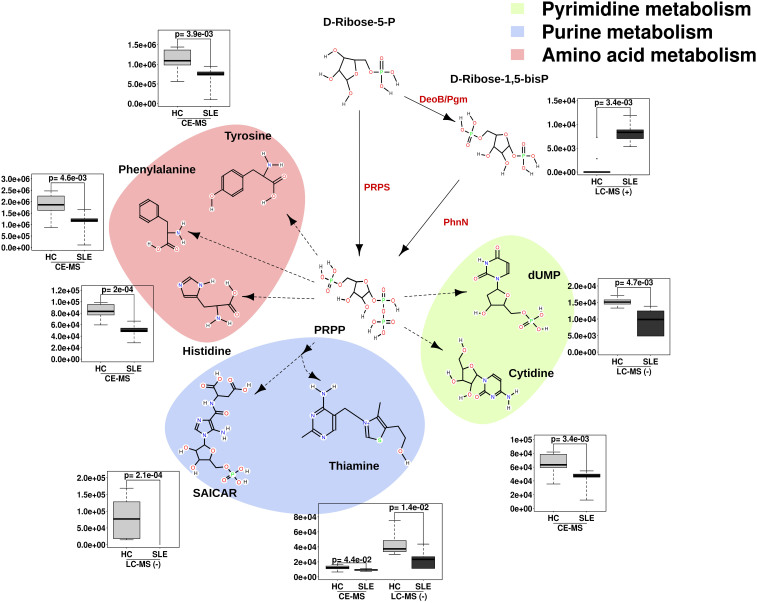
Box plots of key metabolite abundance levels in SLE patients compared with HC controls. The data and the statistical significances were extracted from the data presented in Supplementary Table 5. The data are presented in the context of the KEGG metabolism pathways and indicate the connection to each of the chemical species.

Significantly increased levels of ribose-1,5-bisphosphate (R1,5-dP; 629.8-fold increase; *p* = 0.0034) were also observed in SLE patients compared with HC subjects (Figure 3). This chemical species is an intermediate in the production of 5-phospho-alpha-D-ribose-1-diphosphate (PRPP), which is required for *de novo* purine and/or pyrimidine biosynthesis and the synthesis of amino acids such as histidine, tyrosine, and phenylalanine. In fact, an absence of 1-(5′-phosphoribosyl)-5-amino-4-(N-succinocarboxamide)-imidazole (SAICAR) (*p* = 0.0002) and slightly reduced production levels of thiamine (1.9-fold reduction; *p* < 0.042), dUMP (1.7-fold reduction; *p* = 0.005), cytidine (1.5-fold reduction; *p* = 0.0003), histidine (1.7-fold reduction; *p* = 0.0002), tyrosine (1.6-fold reduction; *p* = 0.004), and phenylalanine (1.7-fold; *p* = 0.005) were observed in SLE patients compared with healthy controls; note that the production of these chemical species depends on the PRPP concentration.

**Figure 4:**
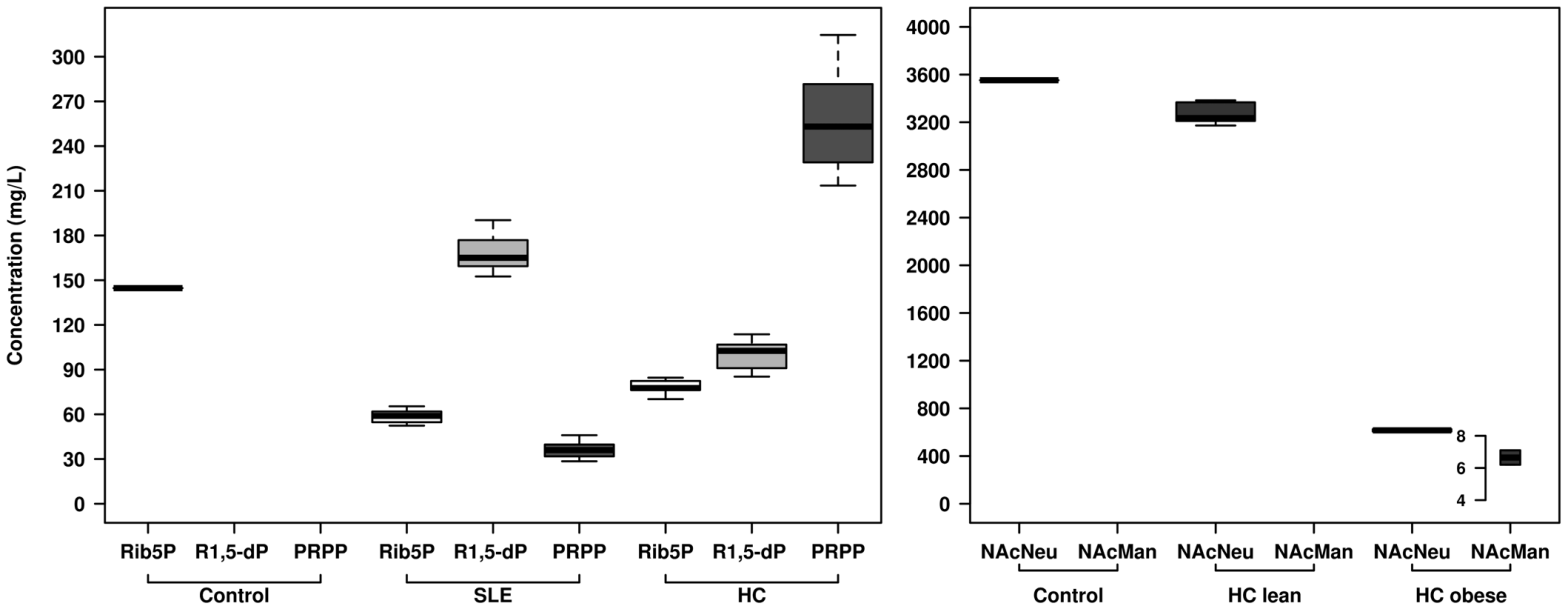
Box plots of chemical species concentrations in reaction tests containing Rib5P (Panel A) and sialic acid (Panel B). The corresponding peak areas were integrated using MassHunter Quantitative Analysis (B.05.00, Agilent). The final concentration of each analyte per sample was calculated using the interpolation of each peak area in the corresponding calibration curve. Reaction conditions were as follows: 1 mL of 50 mM HEPES pH 7.0, 0.1 mg of total protein, and 0.15 mg of Rib5P (Panel A) or 3.5 mg of sialic acid (Panel B). Reactions were incubated for 15 min at 25°C, after which substrate conversion was examined by LC-QTOF-MS using the conditions described in the Methods section. All SLE and HC samples were tested for substrate conversion in Panel A, whereas data for only the lean and obese HC subgroups are shown in B. Reactions without proteins but with Rib5P or sialic acid were used as controls, and the initial concentrations are shown for comparative purposes.

We reasoned that the decreased activity of R1,5-dP-modifying enzymes may lead to an accumulation of this substrate in SLE patients; this in turn may result in reduced production of metabolites in the consequent metabolic steps. This was further confirmed by targeted metabolomics where the extension of the biochemical production of R1,5-dP from its reaction substrate ribose-5-phosphate (Rib5P) as well as the consequent conversion to PRPP (see Figure 4) was evaluated using microbiotal protein extracts. Indeed, the following four presumptive enzymes are implicated in these transformations: *i*) phosphopentomutase (DeoB) and ribose 1,5-bisphosphokinase (PhnN) transforming Rib5P to R1,5-dP; *ii*) phosphoglucomutase (Pgm) metabolizing R1,5-dP to PRPP; and *iii*) ribose-phosphate pyrophosphokinase (PrsA protein), which is implicated in the direct transformation of Rib5P to PRPP. For this transformation, microbial protein extracts from each of the SLE and HC subjects were obtained as previously described^16^ for an activity test using a solution containing Rib5P (see Methods section). The extent of Rib5P transformation and the presence of the R1,5-dP and PRPP reaction products were quantified by LC-QqQ-MS (Figure 4). At the end of our assay, transformation of Rib5P was demonstrated to a similar extent in both groups (54 to 40% residual concentration). A higher concentration of R1,5-dP (1.7-fold) and a significantly lower concentration of PRPP (7.0-fold) were observed in SLE patients compared to HC subjects (Figure 4). This confirms that the accumulation of R1,5-dP in SLE patients is most likely due to a lower level of Pgm activity involved in the transformation of Rib5P to PRPP and not to the increased level of DeoB activity that controls R1,5-dP biosynthesis from Rib5P.

Finally, we further noted that SLE patients accumulated mesoporphyrin IX (*p* < 0.0008) and protoporphyrin IX (*p* = 0.0004), which were absent in HC subjects (Supplementary Table 5). The fact that mesoporphyrin IX is an inhibitor of heme synthesis and ferrochelatase activity is consistent with the accumulation of protoporphyrin IX due to the presumptive inhibition of HemH proteins^27^. We suggest that SLE most likely decreases the iron uptake capacity of the gut microbiota and may also inhibit heme synthesis. In agreement with this, the serum ferritin level in SLE patients (*n* = 18) was approximately 1.5-fold lower (according to mean values) than in HC subjects (*n* = 17) (Supplementary Table 3), and thus a relationship between the deficiency in microbiotal iron uptake observed using a metabolome-wide scan in SLE patients and a lower level of serum ferritin could be suggested.

### Higher BMIs in healthy controls promote the presence of bacteria possessing the sialic acid catabolic pathway

Only 572 of 134,312 (or 0.43% of the total) mass features were statistically (*p* < 0.05) different between healthy controls with high and low BMI values (Figure 1). Therefore, when both groups were compared, the data indicated that the impact of BMI on the gut microbiotal metabolite-wide flux distribution and metabolism itself was moderately low. However, among differences in other chemical species (Supplementary Table 5), an absence of N-acetylneuraminate (*p* = 9.65e^−7^; Supplementary Table 5) was strongly associated with HC subjects with BMIs ≥ 25.24 kg/m^2^ (sub-group “High BMI” in Figure 1). We reasoned that the activities of N-acetylneuraminate lyases, encoded by the sialic acid catabolic gene *nah*A^28^, which remove a pyruvate from N-acetylneuraminate as a first step in the catabolism of sialic acid, should be strongly depleted in lean individuals (here, BMI ≤ 24.83 kg/m^2^). This may result in the accumulation of sialic acid when compared with overweight or obese individuals (sub-group “High BMI”).

To confirm this, the transformation of sialic acid into its corresponding product N-acetyl-D-mannosamine was further examined using a target metabolomics approach, in which the conversion of sialic acid was followed using microbiotal protein extracts (see Methods). The results revealed that lean HC patients were not able to metabolize sialic acid, although it was transformed (only 13.8% residual concentration at the end of the assay) to N-acetyl-D-mannosamine in obese HC subjects (Figure 4). This confirms that the catabolism of sialic acid may be strongly diminished in lean individuals, most likely due to the absence or lower activity level of NahA proteins.

Taken together, it is plausible that individuals from both high and low BMI sub-groups may possess colonic bacteria that have the capacity to liberate sialic acid from the mucosa and transport it to bacterial cells. However, individuals with high BMI (here, ≥25.24 kg/m^2^) may have an additional genomic complement for the sialic acid catabolic pathway (i.e., bacteria that produce NahA) that enables further metabolism of sialic acid, whereas colonic bacteria from individuals with low BMI (here, ≤24.83 kg/m^2^) may be unable to catabolize sialic acid. Thus, sialic acid tends to accumulate in bacterial cells. This was confirmed by biochemical tests and target metabolomics analyses. These data suggest that BMI (e.g., overweight/obesity) may not alter mucosal carbohydrate bioavailability but rather alters how liberated sugars are catabolized. We speculate that community members in individuals with high BMIs may efficiently consume mucosal carbohydrates, which in turn may induce growth and self-promoting host inflammation compared with individuals with low BMI. Consistent with this hypothesis, it has been reported recently that bacteria and pathogens that are unable to catabolize sialic acid exhibit impaired expansion^28^.

## Discussion

We have demonstrated for the first time that the gastrointestinal microbiota can be affected by immune factors. This association was found at the level of the metabolite landscape of gut microbiota (Figure 1) but not at the level of bacterial composition (Figure 2), which suggests that SLE can influence the heterogeneous species inhabiting the gut in such a concerted way that a distinctive metabolic pattern arises. These results demonstrate that deficiencies in the chemical species mediating cell signaling and regulation are among the major effects of SLE. We speculate that lowering quorum sensing, cell-to-cell communication and cell wall biosynthesis, which are known to be of global importance in microbial ecosystems^19^,^20^,^21^,^22^,^23^,^24^, may be partially responsible for the concerted mechanism inducing these common metabolic patterns. Such alterations may also act as disease factors by promoting the immune response, as has been suggested in the case of arthritis^24^,^25^. These alterations may also cause alterations in specific cell-critical systems, such as nucleotide biosynthesis, iron uptake and heme synthesis, without substantial loss of metabolic robustness. However, further experimental evidence is needed to confirm the cause and effect relationship between the altered metabolites and the underlying SLE disease.

We also found that BMI has an effect at the level of the metabolite landscape but not at the level of the microbiota composition based on 16S rRNA gene survey. Interestingly, such changes were only noticeable in healthy controls. The impact of BMI on metabolism itself is also limited, and only select robust effects on the catabolism of sialic acid were revealed. The fact that no effect on the production of regulatory/signaling molecules and cell wall synthesis was observed in HC individuals suggests that different mechanisms may be responsible for generating the distinct metabolic patterns of SLE and HC intestinal microbiota and that a regulatory/signaling response may be one of the major causes linking the altered microbial metabolites and SLE disease, where BMI did not have an effect.

Together, the evidence generated in this study demonstrates that the gut microbiota functionality can be affected by immune and weight factors. Also, it demonstrates that metabolome-wide assessments may be a better indicator than 16S rRNA survey to enable not only the segregation of different diseases and disorders (here, SLE and overweight/obesity) but also the ranking of the effects of the disease/disorder on microbiotal metabolism. As an example, we demonstrated that an immune response, exemplified by SLE, is a dominant factor compared to obesity in controlling the metabolism of the intestinal microbiota. We believe that these findings, for which no previous evidence exists in the scientific literature, potentially open new research avenues for investigating the response mechanisms of human gut microbiota to a single or collective immune, genetic, pathogenic, and dietary pressures, and more importantly, the interaction and relative clinical importance of each of these factors for the progression of different diseases and predispositions to metabolic dysfunctions, such as metabolic syndrome in SLE patients^13^.

Only 955 of 134,312 (or 0.72% of the total) and 572 of 134,312 (or 0.43% of the total) mass features were found to significantly differ between the SLE and HC groups and between HC subjects with high and low BMI values, respectively. It is therefore important to evaluate whether or not such subtle differences can be considered within a common range. It is worth noting, however, that no report to date has described the metabolomic profiling of either bacterial fecal extracts or fecal fluids from patients with SLE; therefore, little is known about whether the observed differences between SLE and HC are within a common range. In the case of subjects that are discordant for weight, few examples exist in the literature that have examined fecal fluid metabolomes. Thus, it should be highlighted that a recent report examining the cecal metabolome revealed that only 65 out of a total of 10,515 mass signals (or 0.7% of the total) were significantly associated with a high-fat diet^29^. In a different study, only 22 fecal metabolites have been shown to be differentially produced in monozygotic twin pairs that were discordant for weight^18^. In cases of other pathophysiologies, using metabolite profiling of fecal fluids, it was found that only: *i*) 18 fecal metabolites allowed discrimination between ulcerative colitis and irritable bowel syndrome and healthy control patients^30^; *ii*) 99 allowed discrimination of humanized and gnotobiotic mice, even though they possess quite distinct microbiota (85% of genera and microbial species are different)^31^; *iii*) 43 metabolites were found to differ when comparing human, mouse and rat fecal metabolomes^32^; and *iv*) 22 metabolites allow the segregation of patients with colorectal cancer compared to healthy adults^33^. Therefore, based on bibliographic records in the specialized literature, the subtle differences associated with SLE or BMI reported in the present study can be considered within a common range observed for, or even few times higher than, those observed for reported pathophysiologies.

The further question that arises is whether these subtle differences in gut microbiota functionality are sufficient to have physiological implications. Based on the data reported herein, it is plausible that only selective effects in a number of key metabolites with major biological relevance/significance may be sufficient to induce gut homeostasis or alterations in gut microbiota functionality, even though the impact on the global metabolome is moderate, regardless of the heterogeneities at the population level. The deficiencies observed in chemical species participating in, for example, quorum sensing, cell-to-cell communication and cell wall biosynthesis, which are known to be of global importance in microbial ecosystems, agree with this hypothesis. Having said that, it should be noted that in many cases, minor differences, e.g., at the population level, have been demonstrated to induce strong physiological changes. As an example, it has recently been demonstrated that one or two strains are sufficient to drive major changes in gastrointestinal and host (mouse) metabolic profiles where up to 10^12^ microbial cells or more than 500 species may coexist^31^.

The effects of various pathophysiologies in the human gut metabolome have been previously examined^18^,^29^,^30^,^31^,^32^,^33^. However, no clear associations between fecal fluid metabolome patterns and individual pathophysiologies (e.g., weight gain or the presence of a disease) were previously observed; this was mainly due to the large inter-individual variation. For example, the examination of fecal metabolomes from at least 10 obese mice (body weight change: from 2 to 8 grams) revealed heterogeneous distributions, and no clear clusters were visible at the BMI level^29^. Such inter-individual variation was not observed in this study, as mass signals within grouped subjects (SLE patients and HC subjects with “low” or “high” BMI) were highly similar regardless of age, disease duration, dietary intake, lifestyle-related factors or medical history. One of the major differences from previous studies is that herein we focused on the isolation of metabolites from microbes isolated from stool material followed by a metabolome-wide scan, rather than examining total fecal fluids. In relation to this examination, the microbiota is the central bioreactor of the gastrointestinal tract, and a dynamic interplay exists with the host and the environment^7^. As a result of metabolic actions and environmental inputs, the gut environment and, in turn, the fluid fecal material contains a complex mixture of metabolites provided through the diet, the host and intestinal bacteria. Such complex mixtures are commonly investigated in metabolomics studies^18^,^29^,^30^,^31^,^32^,^33^. Metabolites from intestinal bacteria, rather than dietary and host metabolites, are required to maintain and repair the large intestine and to support human health^34^. Therefore, any knowledge related to metabolites that are directly produced or adsorbed (from environmental inputs or the host) by gut microbes, not those present in complex whole fecal fluids, may be of relevance not only for investigating what is happening throughout the gut but also for determining their role in pathophysiologies and human health. We believe this investigation will provide information that can be directly linked to complementary microbial data, i.e., 16S rRNA gene profiles, which is difficult to achieve otherwise if non-microbial metabolites (from the environment or host), which are commonly considered when working with whole fecal material, are investigated. In the present study, metabolites from intestinal bacteria have been shown to be good indicators of gut microbiota functionality under various pathophysiologies, and they may be more effective than fecal fluids as a read-out of pathophysiologically induced alterations. Note that our study relates to metabolite levels inside gut bacterial cells, which may have a different meaning than those in plasma and, to some extent, in fecal fluids.

Finally, further research is required of the mechanisms that generate the distinct and robust gut microbial metabolic profiles discussed herein. This investigation will provide a deeper view on *what the microbiota do* rather than *who they are*. We hypothesize that new reliable clinical information and explanatory and mechanistic plausibility for these associations as well as new sensitive, predictive disease biomarkers of clinical relevance may arise when the microbiotal metabolite landscape rather than heterogeneous species gut composition are investigated.

## Methods

### Chemicals and reagents

The following reagents were used: acetonitrile (HPLC-MS grade, Sigma-Aldrich, Taufkirchen, Germany), formic acid (MS grade, Sigma-Aldrich, Steinheim, Germany), L-methionine sulfone (Sigma-Aldrich, Taufkirchen, Germany), sodium hydroxide (Panreac, Montcada I Reixac, Spain) and ammonia (Panreac, Castellar del Vallès, Barcelona, Spain). For reference masses, purine and hexakis(1H,1H,3H-tetrafluoropropoxy)phosphazine (HP) from Agilent (API-TOF reference mass solution kit) were used. All solutions were prepared using MilliQ® water (Millipore, Billerica, MA, USA).

### Sample treatment for metabolite isolation

Fresh stool samples were collected from each subject, frozen immediately, and stored at −80°C until they were processed. A total of 34 samples were metabolome-typed using a combination of untargeted mass spectrometry and two different and complementary separation techniques (liquid chromatography-mass spectrometry [LC-ESI-QTOF-MS] and capillary electrophoresis-mass spectrometry [CE-TOF-MS]). To facilitate this analysis, microbial cells were separated from the fecal material, and the total microbial metabolites were extracted from equal amounts of microbial cells per sample by adapting a previously reported method^4^ and including a two-step extraction method that was shown to produce the optimal extraction efficiency for both polar and hydrophobic metabolites.

Briefly, microbial cells were separated from the fecal matrix by mixing 0.4 g of fecal sample with 1.2 mL of phosphate-buffered saline (PBS) solution (1:3 w/v feces to PBS ratio); following re-suspension (by 1 min of vigorous vortexing), the samples were then centrifuged at 1,000 *g* at 4°C for 1 min to remove fecal debris. The supernatant (0.6 mL) was transferred to a 2-mL Eppendorf tube and centrifuged at 13,000 *g* at 4°C for 5 min to pellet the cells. Immediately after isolating the microbial cells, the cells were used for MeOH extraction by adding 1.2 mL of cold (−80°C) HPLC-grade MeOH. The samples were then vortex-mixed (for 10 s) again and sonicated for 30 s (in a Sonicator® 3000; Misonix) at 15 W in an ice cooler (−20°C). This protocol was repeated twice more with a 5-min storage at −20°C between each cycle, and the final pellet was removed following centrifugation at 12,000 *g* for 10 min at 4°C. Immediately after the MeOH extracts were obtained, the MeOH solution was stored at −80°C, and the remaining cell pellet was re-suspended in 1.2 mL of cold (4°C) HPLC-grade H_2_O and subjected to 3 cycles of sonication for 20 s (in a Sonicator® 3000; Misonix) at 15 W in ice water. The samples were incubated on ice for 2 min between cycles. The final pellet was removed following centrifugation at 12,000 *g* for 10 min at 4°C. Immediately after the H_2_O and MeOH extracts were obtained, a mixture was prepared by combining equal amounts (1 mL) of each of the extracts. Once prepared, the final solution was stored in 20-mL penicillin vials at −80°C for use.

Two hundred microliters of the cellular extracts were mixed with 200 μL of acetonitrile to precipitate the proteins. This solution was then centrifuged at 13,000 rpm at 4°C for 10 min to separate any solid impurities. The supernatants were removed and filtered through 0.2-μm nylon syringe filters. Then, 100 μL were transferred to analytical vials for LC-MS analysis. For CE-MS analysis, 70 μL of filtered extract were evaporated to dryness using a Speedvac Concentrator, and each sample was then reconstituted in 70 μL of MilliQ® water containing an internal standard (0.2 mM L-methionine sulfone) and 0.1 M formic acid.

### Preparation of quality controls (QCs) for metabolomic fingerprinting

Because the samples interact during the separation technique and MS, it is crucial to employ quality controls (QCs) during LC-MS and CE-MS to ensure analytical reproducibility. Indeed, QC samples are required at the beginning of the sequence to stabilize the system and throughout the analytical runs at periodic intervals of time to monitor variations in signal across time^35^. QC samples were prepared independently for LC-MS and CE-MS by pooling and mixing equal volumes of each sample. After gently vortexing, the mix was also filtered and subsequently transferred to an analytical vial.

### Metabolomic fingerprinting by LC-ESI-QTOF-MS

The metabolic profile was achieved using a liquid chromatography system consisting of a degasser, a binary pump, and an autosampler (1290 infinity, Agilent). Samples (0.5 μL) were applied to a reversed-phase column (Zorbax Extend C_18_ 50 × 2.1 mm, 3 μm; Agilent), which was maintained at 60°C during the analysis. The system was operated at a flow rate of 0.6 mL/min with solvent A (water containing 0.1% formic acid) and solvent B (acetonitrile containing 0.1% formic acid). The gradient was 5% B (0–1 min), 5 to 80% B (1–7 min), 80 to 100% B (7–11.5 min), and 100 to 5% B (11.5–12 min). The system was finally held at 5% B for 3 min to re-equilibrate the system (15 min of total analysis time). Data were collected in positive and negative ESI modes in separate runs using QTOF (Agilent 6550 iFunnel). Analyses were performed in both positive and negative ion modes. The positive mode was operated in full-scan mode from *m/z* 50 to 1000. The capillary voltage was 3000 V with a scan rate of 1.0 spectrum per second. The gas temperature was 250°C, the drying gas flow was 12 L/min and the nebulizer was 52 psi. The MS-TOF parameters were as follows: fragmentor, 175 V; skimmer, 65 V; and octopole radio frequency (OCT RF Vpp) voltage, 750 V. The negative ion mode was operated in full-scan mode from *m/z* 50 to 1100. The capillary voltage was 3000 V with a scan rate of 1.0 spectrum per second. The gas temperature was 250°C. The drying gas flow was 12 L/min, and the nebulizer was 52 psi. The MS-TOF parameters included the following: fragmentor, 250 V; skimmer, 65 V; and octopole radio frequency voltage, 750 V. During the analyses, two reference masses were used: 121.0509 (detected *m/z* [C_5_H_4_N_4_ + H]^+^) and 922.0098 (detected *m/z* [C_18_H_18_O_6_N_3_P_3_F_24_ + H]^+^) in positive mode and 112.9855 (detected *m/z* [C_2_O_2_F_3_-H]^−^) and 1033.9881 (detected *m/z* [C_18_H_18_O_6_N_3_P_3_F_24_ + TFA-H]^−^) in negative mode. The references were continuously infused into the system, enabling constant mass correction. Samples were analyzed in randomized runs, during which they were incubated in an autosampler at 4°C. The analytical runs for both polarities were set up starting with the analysis of ten QCs followed by the samples; a QC sample was injected in between blocks of five samples until the end of the run.

### Metabolomic fingerprinting by CE-TOF-MS

A capillary electrophoresis apparatus (7100 Agilent) coupled to a TOF Mass Spectrometer (6224 Agilent) was employed. The CE mode was controlled by ChemStation software (B.04.03, Agilent), and the MS mode was controlled by Mass Hunter Workstation Data Analysis (B.02.01, Agilent). The separation occurred in a fused-silica capillary (Agilent: total length, 100 cm; internal diameter, 50 μm). All separations were performed in normal polarity with a background electrolyte containing 0.8 M formic acid in 10% methanol (*v/v*) at 20°C. New capillaries were pre-conditioned with a flush of 1.0 M NaOH for 30 min followed by MilliQ® water for 30 min and the background electrolyte for 30 min. Prior to each analysis, the capillary was conditioned with a flush of background electrolyte for 5 min. The sheath liquid (6 μL/min) was MeOH:H_2_O (1:1) containing 1.0 mM formic acid with two references masses of *m/z* 121.0509 ([C_5_H_4_N_4_ + H]^+^) and *m/z* 922.0098 ([C_18_H_18_O_6_N_3_P_3_F_24_ + H]^+^), which enabled correction and higher mass accuracy during MS. Samples were hydrodynamically injected at 50 mBar for 50 s. The stacking was performed by applying the background electrolyte at 100 mBar for 10 s. The separation voltage was 30 kV, and the internal pressure was 25 mBar; the analyses were performed within 35 min. The MS parameters included the following: fragmentor, 100 V; skimmer, 65 V; octopole, 750 V; drying gas temperature, 200°C; flow rate, 10 L/min; and capillary voltage, 3500 V. Data were acquired in positive mode with a full scan from *m*/*z* 85 to 1000 at a rate of 1.41 scan/s. The analytical run started with an analysis of five QCs followed by the samples; a QC sample was injected in between blocks of five samples until the end of the run.

### Metabolomic data treatment

The Feature Extraction tool in the Mass Hunter Qualitative Analysis software (B.05.00, Agilent) was used. The alignment of the raw data was performed using MassProfiler Professional software (B.12.01, Agilent). The variables were then filtered. Data present in at least 50% of the QCs, with coefficients of variation less than 30% across the QCs, were selected, and models were subsequently built using SIMCA-P+ software (12.0.1.0, Umetrics; Figure 1). Based on the PCA and the patient's BMI, sample HC4 was rejected prior to statistical analysis. Subsequently, any missing values were replaced by the mean (if the variable was present in more than 2/3 of the samples per group) or by half of the minimum value (if the variable was present in 1/3 to 2/3 of the samples per group). Missing variables that were present in less than 1/3 of samples per group were denoted as zero. Finally, groups were compared in pairs (SLE vs. HC and HCh [HC with high BMI] vs. HCl [HC with low BMI]) using Mann-Whitney *U* tests or *t*-tests followed by Bonferroni corrections to minimize false positives (corrected *p* value ≤0.05; MATLAB 7.10.0.499). The resulting list of accurate masses that significantly differed between groups was searched using the CEU Mass Mediator search tool (http://biolab.uspceu.com/mediator; error ± 5 ppm). This procedure was performed independently for each analytical platform.

Note that the robustness of the analytical procedure was demonstrated by the tight clustering of the QCs (Figure 1), showing that the separation between groups was due to actual biological variability and was not random.

### 16S rRNA microbiota analysis

The QIIME software suite was used to construct a UniFrac similarity matrix of all 16S rRNA profiling samples included in this study based on their operational taxonomic unit (OTU) profiles^11^. This similarity matrix was processed to obtain a three-dimensional principal coordinate analysis (PCoA) where the percentages shown along the axes represent the proportion of dissimilarities between samples captured by the axes (Figure 2). The raw sequences reported in this article have been deposited in the NCBI Short Read Archive (SRA) (study accession number: SRP028162)^11^.

### Biochemical tests by targeted metabolomics

Protein extracts from each of the SLE patients and HD subjects were obtained and used for activity tests as previously described^17^. The extent of transformation of Rib5P to R1,5-dP and to PRPP was quantified in a solution containing 1 mL of 50 mM 4-(2-hydroxyethyl piperazine-1-ethanesulfonic acid (HEPES), pH 7.0, 0.15 mg/mL ribose-5-phosphate and 0.1 mg/mL total protein. Similarly, transformation of sialic acid (3.5 mg/mL) to N-acetyl-D-mannosamine was performed under similar conditions. Reactions were kept for a total time of 15 min at 25°C, and substrate conversion was examined by LC-QqQ-MS as described below. Reactions without proteins and substrates were used as controls; they were subsequently treated in the same manner as samples.

#### Sample treatment

Prior to analysis, the samples were diluted 1:3 with MilliQ® water (dilution factor 4), and a pool of samples was prepared by extracting 10 μL from each vial. Independently, a mix of standards (Rib5P, R1,5-dP, PRPP, N-acetyl-neuraminic acid and N-acetyl-D-mannosamine and N-acetyl-neuraminic acid; all from Sigma Chemical Co., St. Louis, MO) was prepared from independent solutions of each compound, with a final concentration of 10 ppm (mg/L) in MilliQ® water, and subsequently diluted to 7.5, 5, 2.5 and 1 ppm (mg/L).

#### LC-QqQ-MS

Each analysis was achieved using a liquid chromatography system consisting of a degasser, binary pump and autosampler (1290 Infinity, Agilent Technologies, Santa Clara, CA, USA) coupled to a triple quadrupole mass spectrometer (6460, Agilent Technologies). A Kinetex® HILIC column (150 × 2.1 mm, 2.6 μm, Phenomenex, Torrance, CA, USA) was maintained at 50°C during the analysis. The system was operated at a flow rate of 0.6 mL/min with solvent A (water with 5 mM of ammonium formate, pH 6.8) and solvent B (acetonitrile). The gradient was as follows: 75% B (0–0.5 min), 75 to 0% B (0.5–5 min), 0% B (5–6 min) and 0 to 75% B (6–7 min), keeping the re-equilibration at 75% B for 4 min (11 min of total analysis time). Data were collected in negative MRM mode. The MS-ESI parameters were optimized as follows: capillary voltage, 2500 V; gas temperature, 200°C; drying gas flow, 8 L/min; nebulizer, 48 psi; and nozzle voltage, 0 V. The MS-QqQ parameters were as follows: fragmentor, 116 V; dwell time, 20 ms; and cell accelerator voltage, 7 V. The *m/z* quantification transition for each compound was as follows: Rib5P (229-97), R1,5-dP (309-97), PRPP (388.9-79), N-acetyl-mannosamine (220.1-59.1) and N-acetyl-neuraminic acid (308.1-87). The analytical run started with the analysis of ten injections of the pool to equilibrate the chromatographic system and was followed by the samples in a randomized order. Samples were maintained at 4°C throughout the run, and 20 μL of each sample were injected.

### Study approval

Ethical approval for this study (reference code AGL2010-14952; grant title “Towards better understanding of gut microbiota functionality in some immune disorders”) was obtained from the Bioethics Committee of CSIC and from the Regional Ethics Committee for Clinical Research (*Servicio de Salud del Principado de Asturias*) in compliance with the Declaration of Helsinki. All determinations were performed with fully informed written consent from all participants involved in this study. All experiments were performed in accordance with approved guidelines and regulations.

## Author Contributions

The study was conceived by M.F. and A.Ma. All authors contributed to the data collection. A.H., D.R., M.M.-M. and M.F. performed the experiments, and R.B. contributed to data analysis. Data interpretation and manuscript preparation were performed by D.R. and M.F. P.L., A.C., S.G. and Ana.S. provided the fecal material and clinical records. B.S. provided biodiversity input. C.M. and M.V. provided 16S rRNA and bioinformatic data analysis. C.B. provided analytic and intellectual input on the metabolome data. Ant.S and A.Mo. provided intellectual input. All authors have critically reviewed and edited the manuscript and have approved its publication.

### Additional information

**Nucleotide sequence accession number** The NCBI Short Read Archive (SRA) accession numbers described in this study are SRP028162.

## Supplementary Material

Supplementary Information Supplementary Tables 1-3

Supplementary Information Supplementary Table 4

Supplementary Information Supplementary Table 5
