# Supplementary material for: Ranking the impact of human health disorders on gut metabolism: Systemic lupus erythematosus and obesity as study cases
Source: Sci Rep. 2015 Feb 6;5:8310. doi: 10.1038/srep08310 (PMC4319156; doi:10.1038/srep08310)
Supplement: Supplementary Information — Supplementary Tables 1-3 [file srep08310-s1.doc]

**Ranking the impact of human health disorders on gut metabolism: Systemic lupus erythematosus and obesity as study cases**

David Rojo, Arancha Hevia, Rafael Bargiela, Patricia López, Adriana Cuervo, Sonia González, Ana Suárez, Borja Sánchez, Mónica Martínez-Martínez, Christian Milani, Marco Ventura, Coral Barbas, Andrés Moya, Antonio Suárez, Abelardo Margolles, Manuel Ferrer

**Supplementary table legends**

**Supplementary Table 1** Demographics and clinical features of the SLE patients.

**Supplementary Table 2** Summarized general and disease characteristics of the SLE patients and HC individuals. Full details for each subject are provided in Supplementary Table 3.

**Supplementary Table 3** Detailed characteristics of the SLE patients and HC individuals. BMI values are included. Numerical code for column “Health”: 0, excellent; 1, good; 2, normal; 3, regular; 4, bad. Numerical code for column “Smoke”: 0, no smoke; 1, smoker; 2, ex-smoker.

**Supplementary Table 4** List of raw and statistically (designated as ST) significant masses identified and quantified in a metabolome-wide scan of gut microbiota. For differential quantitative metabolomics, we compared the metabolomes of samples by evaluating peak areas from chromatographic peaks. A list of masses identified by LC-MS using positive and negative polarities and CE-MS following alignment are presented for SLE vs. HC and for HC low (HCl) vs. high (HCh) BMI. The technique (LC-MS positive (+) or negative (-) mode or CE-MS), mass error (in ppm), retention time (RT; as ppm@RT), the *p* value calculated using Mann-Whitney *U* test or *t*-test (denoted “*p* *t*-test or Mann-Whitney *U* test”) followed by Bonferroni corrections (designated “p Bonf), and the abundance level per sample (SLE or HC) and per group of samples (average [X] for “HC”, “SLE”, “HCh”, or “HCl” groups) are shown. Statistically significant differences (*p* values) per metabolite identified, and pairwise comparisons among mean values (abundance levels) are provided. Panel abbreviations and content as follows: LC+ raw, LC- raw, and CE raw, list of masses identified in LC-MS using positive (+) and negative (-) polarities and CE-MS, respectively, following alignment; LC+ ST SLE vs. HC, LC- ST SLE vs. HC, and CE ST SLE vs. HC, list of differential/statistically significant masses identified in LC-MS using positive and negative polarities and CE-MS, respectively, in the SLE patients compared with HC subjects; LC+ ST HCh vs. HCl, LC- ST HCh vs. HCl, and CE ST HCh vs. HCl, list of differential/statistically significant masses identified in LC-MS using positive and negative polarities and CE-MS, respectively, in the HCh subjects compared with HCl subjects.

**Supplementary Table 5** List of putatively identified (ID) masses of molecules that achieved statistical criteria (Supplementary Table 4) and were responsible for samples/groups separations. The technique (LC-MS positive (+) or negative (-) mode or CE-MS), experimental mass (designated “Mass”), retention time (RT), theoretical mass (designated “Designated mass”), mass error (in ppm), putative name and formula, *p* values calculated using Mann-Whitney *U* test or *t*-test (denoted “*p* *t*-test or Mann-Whitney *U* test”) followed by Bonferroni corrections (designated “p Bonf), and the abundance level per sample (SLE or HC) and per group of samples (average [X] for “HC”, “SLE”, “HCh”, or “HCl” groups) are presented. Panel abbreviations and content as follows: LC+ ID SLE vs. HC, LC- ID SLE vs. HC, and CE ID SLE vs. HC, list of putatively identified masses in LC-MS using positive (+) and negative (-) polarities and CE-MS, respectively, that significantly differed in the SLE patients compared with HC subjects; LC+ ID HCh vs. HCl, LC- ID HCh vs. HCl, and CE ID HCh vs. HCl, list of putatively identified masses in LC-MS using positive and negative polarities and CE-MS, respectively, that were significantly different in the HCh subjects compared with HCl subjects.

**Supplementary Table 1** Demographics and clinical features of the SLE patients.

| **Total SLE patients*** | **n=18** |
| --- | --- |
| Age at diagnosis, median yearts (IQR) | 35.00 (15.00) |
| Disease duration, median years (IQR) | 7.00 (9.00) |
| Clinical manifestations, n (%) |  |
| Malar rash | 9 (50.0) |
| Discoid lesions | 6 (33.3) |
| Photosensitivity | 14 (77.8) |
| Oral ulcers | 9 (50.0) |
| Arthritis | 10 (55.6) |
| Serositis | 3 (16.7) |
| Renal disorder | 3 (16.7) |
| Neurological disorder | 0 (0.0) |
| Haematological disorder | 9 (50.0) |
| Anti-dsDNA, n (%) | 9 (50.0) |
| Titer, median U/ml (IQR) | 10.35 (36.45) |

*dsDNA: double stranded DNA; IQR: interquartile range

**Supplementary Table 2** Summarized general and disease characteristics of the SLE patients and HC individuals. Full details for each subject are provided in Supplementary Table 3.

|  | **SLE patients**  **(n=18)** | **HC subjects**  **(n=17)** |
| --- | --- | --- |
| Female sex (%) | 100 | 100 |
| Age (year) (mean ± sd) | 49.1 ± 9.7 | 48.5 ± 8.0 |
| Smokers (%) | 29 | 24 |
| Habitual alcohol consumers (%) | 35 | 59 |
| Regular physical activity (%) | 35 | 59 |
| Compsumption of fermented foods (%) | 12 | 35 |
| Use of vitamins and mineral supplements (%) | 18 | 41 |

**Supplementary Table 3** Detailed characteristics of the SLE patients and HC individuals. BMI values are included. Numerical code for column “Health”: 0, excellent; 1, good; 2, normal; 3, regular; 4, bad. Numerical code for column “Smoke”: 0, no smoke; 1, smoker; 2, ex-smoker.

**Supplementary Table 3 cont.** Detailed characteristics of the SLE patients and HC individuals. BMI values are included. Numerical code for column “Health”: 0, excellent; 1, good; 2, normal; 3, regular; 4, bad. Numerical code for column “Smoke”: 0, no smoke; 1, smoker; 2, ex-smoker.

**Supplementary Table 3 cont.** Detailed characteristics of the SLE patients and HC individuals. BMI values are included. Numerical code for column “Health”: 0, excellent; 1, good; 2, normal; 3, regular; 4, bad. Numerical code for column “Smoke”: 0, no smoke; 1, smoker; 2, ex-smoker.

**Supplementary Table 3 cont.** Detailed characteristics of the SLE patients and HC individuals. BMI values are included. Numerical code for column “Health”: 0, excellent; 1, good; 2, normal; 3, regular; 4, bad. Numerical code for column “Smoke”: 0, no smoke; 1, smoker; 2, ex-smoker.
